# Opportunities for nutrition in primary care

**DOI:** 10.1017/S1368980019005342

**Published:** 2020-01

**Authors:** Allison Hodge

After being in the Editor-in-Chief role for 12 months I would like to take the opportunity provided in this editorial to thank all the reviewers and members of the editorial board for their work over the year ensuring we continue to publish high quality manuscripts on the diversity of topics that public health nutrition encompasses. Also, the authors who provide the manuscripts and continue to choose PHN over all the other nutrition journals as the place to submit their manuscripts. I wish all readers of PHN and those who have contributed in different ways, all the best for 2020.

Two recent articles in PHN address the issue of nutrition care provision in primary care^(1,2)^. One of these papers reports on a cost-effectiveness analysis of weight-loss dietary counselling by primary care nurses in New Zealand^(1)^, and the other reviewed 16 studies of the behaviour, motivation and opportunity for primary care physicians to provide nutrition care^(2)^. Given the importance of appropriate nutrition in promoting health, expanding the number of health professionals who can deal with nutrition issues is seen as one way of improving population health^(3)^. This editorial will briefly review the related work that has been published in PHN.

As early as 2002, Moore and Adamson, in this journal^(4)^ reported on the results of a 1997 survey by primary care staff (GPs, practice nurses, health visitors, district nurses and dietitians) and their patients, on nutrition interventions. Most of the practitioners demonstrated reasonable knowledge in relation to nutrition and cardiovascular disease and had a positive attitude towards performing nutrition interventions. Agreement with statements regarding the importance of nutrition for health, and the role of primary care workers in nutrition was 99 %. Of more than 2000 patients screened, only 13 % reported having discussed nutrition in the preceding consultation. Both practitioners and patients had raised the issue of diet during consultations, indicating some expectation in patients that this was an appropriate topic to be covered. The study concluded that practitioners knowledge needs to be improved and their advice could be more practical in order to meet the expectations of the 2000 National Service Framework for Coronary heart Disease^(4)^.

A randomised clinical trial of healthy adults in Brazil found that short-term nutritional counselling delivered through primary care was effective in improving behavioural and metabolic parameters^(5)^, but the intervention was conducted by nutritionists and included 3 individual dietary counselling sessions so does not assess the inclusion of dietary advice in other health care consultations.

More recently, Broad and Wallace^(6)^ reviewed nutrition and public health in medical education in the UK. Although there was an expectation that new doctors should understand inter-relationships between nutrition, health and disease, and be competent to manage nutrition and hydration, final year students and new graduates from their medical school did not feel confident in clinical or public health nutrition. A 6-week elective course among a small group of volunteer students improved confidence and knowledge, indicating the potential to improve the situation with a relatively small additional time spent studying nutrition. An inter-professional approach to nutrition education was recommended, promoting teamwork both for learning and implementation.

A commentary^(3)^ on the study by Broad and Wallace^(6)^, noted that although public health nutrition training for doctors was important, there is increasing acknowledgement that all health-care professionals need training in nutrition to supplement the nutrition workforce.

Dean etal.^(7)^ previously pointed out the beneficial reinforcement of recommendations for lifestyle changes if consistent messages are delivered by different health professionals. They describe a process whereby health professionals assess health-related behaviours across smoking, nutrition and physical activity and then initiate intervention or refer to another health professional. Subsequently, different health professionals continue to monitor, provide feedback and refer elsewhere as required. Importantly, this process is integrated, and advice builds over time, rather than being delivered independently in slightly different ways by different health professionals.

Despite considerable interest in nutrition training for medical students and other health professionals, a review of 16 studies into the nutrition care provided by primary care physicians^(2)^, found that participants felt they lacked the capability to provide nutrition care. This review, which was based on the COM-B model considering capability, motivation and opportunity to perform an intervention, in this case nutrition care, reported that motivation varied with characteristics of both the patient and the physician and opportunity was influenced by training and policy from professional and governmental organizations^(2)^. Motivation could be enhanced by educators and mentors reinforcing and modelling nutrition in health care. Better education could improve motivation by informing physicians of the importance of health care and improving their confidence in techniques to promote behavioural change in patients. Policy needs to support the inclusion of nutrition care in general clinical consultations.

We have published a variety of papers supporting the inclusion of nutrition care in primary care, and there are many reasons why this might be a good idea, but to date there has been little attempt to evaluate the benefits of doing so. The last study to mention is a cost-effectiveness analysis of weight loss advice by primary care nurses using New Zealand data^(1)^. The modelling revealed that brief dietary advice for weight loss would generate relatively small benefits and was unlikely to be cost-effective. The intervention modelled included only a 1-hour appointment with a practice nurse and an additional hour of follow-up. This is not consistent with the idea of ongoing advice from different health professionals that has been advocated by Dean etal.^(7)^ and Shrimpton and Blaney^(3)^, but does remind us that it is important to evaluate such initiatives before widespread roll out.

The Need for Nutrition Education/Innovation Programme (NNEdPro) was established in the UK in 2008 to promote nutrition education in health professionals, and now has an international network to further this aim^(8)^. There is likely to be continuing expansion of initiatives to incorporate nutrition care in the roles of health professionals to address the enormous issue of both under- and over-nutrition globally, and this should be seen as an opportunity rather than a threat by nutrition trained health professionals. It will be interesting to read more on this topic in the future.
